# Quantification of Total and Unbound Selinexor Concentrations in Human Plasma by a Fully Validated Liquid Chromatography-Tandem Mass Spectrometry Method

**DOI:** 10.3390/pharmaceutics17070919

**Published:** 2025-07-16

**Authors:** Suhyun Lee, Seungwon Yang, Hyeonji Kim, Wang-Seob Shim, Eunseo Song, Seunghoon Han, Sung-Soo Park, Suein Choi, Sungpil Han, Sung Hwan Joo, Seok Jun Park, Beomjin Shin, Donghyun Kim, Hyeon Su Kim, Kyung-Tae Lee, Eun Kyoung Chung

**Affiliations:** 1 Department of Pharmacy, College of Pharmacy, Kyung Hee University, Seoul 02447, Republic of Korea; sh198410@woosuk.ac.kr (S.L.); u00u96@khu.ac.kr (H.K.); shsonic95@khu.ac.kr (S.H.J.); psjqkr0824@naver.com (S.J.P.); rangers9804@khu.ac.kr (B.S.); waterlion3@khu.ac.kr (D.K.); u00u@khu.ac.kr (H.S.K.); 2 Department of Pharmacy, College of Pharmacy and Research Institute of Pharmaceutical Sciences, Woosuk University, Wanju 55338, Republic of Korea; 3 Department of Regulatory Science, Graduate School, Kyung Hee University, Seoul 02447, Republic of Korea; syang345@khu.ac.kr; 4 Institute of Regulatory Innovation Through Science, Kyung Hee University, Seoul 02447, Republic of Korea; 5 Kyung Hee Drug Analysis Center, College of Pharmacy, Kyung Hee University, Seoul 02447, Republic of Korea; wsshimm@khu.ac.kr (W.-S.S.); sssk2303@khu.ac.kr (E.S.); 6 Department of Pharmacology, College of Medicine, The Catholic University of Korea, Seoul 06591, Republic of Korea; waystolove@catholic.ac.kr (S.H.); mychloe00@gmail.com (S.C.); shan@catholic.ac.kr (S.H.); 7 Hematology Hospital, Seoul St. Mary’s Hospital, The Catholic University of Korea, Seoul 06591, Republic of Korea; imsnake@catholic.ac.kr; 8 Department of Biomedical and Pharmaceutical Sciences, Graduate School, Kyung Hee University, Seoul 02447, Republic of Korea; 9 Department of Pharmacy, Kyung Hee University Hospital at Gangdong, Seoul 05278, Republic of Korea

**Keywords:** selinexor, protein binding, bioanalytical method, LC-MS/MS, validation, human plasma, multiple myeloma

## Abstract

**Background/Objectives:** Selinexor is a selective nuclear-export inhibitor approved for hematologic malignancies, characterized by extensive plasma protein binding (>95%). However, a validated analytical method to accurately measure the clinically relevant unbound fraction of selinexor in human plasma has not yet been established. This study aimed to develop a fully validated bioanalytical assay for simultaneous quantification of total and unbound selinexor concentrations in human plasma. **Methods:** We established and fully validated an analytical method based on liquid chromatography–tandem mass spectrometry (LC-MS/MS) capable of quantifying total and unbound selinexor concentrations in human plasma. Unbound selinexor was separated using ultrafiltration, and selinexor was efficiently extracted from 50 μL of plasma by liquid–liquid extraction. Chromatographic separation was achieved on a C18 column using an isocratic mobile phase (0.1% formic acid:methanol, 12:88 *v*/*v*) with a relatively short runtime of 2.5 min. **Results:** Calibration curves showed excellent linearity over a range of 5–2000 ng/mL for total selinexor (r^2^ ≥ 0.998) and 0.05–20 ng/mL for unbound selinexor (r^2^ ≥ 0.995). The precision (%CV ≤ 10.35%) and accuracy (92.5–104.3%) for both analytes met the regulatory criteria. This method successfully quantified selinexor in plasma samples from renally impaired patients with multiple myeloma, demonstrating potential inter-individual differences in unbound drug concentrations. **Conclusions:** This validated bioanalytical assay enables precise clinical pharmacokinetic assessments in a short runtime using a small plasma volume and, thus, assists in individualized dosing of selinexor, particularly for renally impaired patients with altered protein binding.

## 1. Introduction

Selinexor is an anticancer agent approved for the treatment of multiple myeloma and lymphoma, targeting exportin 1 (XPO1) to selectively inhibit nuclear export [1,2,3]. It blocks nuclear export of tumor suppressor proteins and growth-regulatory proteins, as well as oncogenic mRNAs from the nucleus, ultimately leading to cancer cell death [1,2,3]. Current practice guidelines recommend selinexor in combination with dexamethasone or other anticancer drugs for treating relapsed or refractory multiple myeloma [4]. Recent clinical studies suggest that an oral treatment regimen combining selinexor, lenalidomide, and dexamethasone may be highly beneficial for patients with either relapsed/refractory or newly diagnosed multiple myeloma [5,6]. With convenient oral administration and ongoing evidence of clinical benefit, selinexor use is expected to increase alongside a rising prevalence of multiple myeloma in an aging population [7].

The currently approved selinexor dose for relapsed or refractory multiple myeloma is either 80 mg on days 1 and 3 of each week or 100 mg once weekly, depending on the combination agents within the anticancer regimen [8,9,10,11]. Dosing information is scarce for specific patient populations, such as patients with kidney impairment or liver dysfunction, which are common complications in advanced-stage multiple myeloma. Although recent studies suggested similar pharmacokinetic exposures of selinexor in patients with mild renal or hepatic impairment to those in individuals with normal organ function [12], dosing adjustment might be warranted in older individuals or patients with moderate to severe organ dysfunction, considering the pharmacokinetic profiles of selinexor. Selinexor is extensively bound to proteins with an estimated protein-binding fraction in plasma of >95%, resulting in a large volume of distribution of approximately 133 L [3,13,14]. The estimated half-life of selinexor is 4 to 8 h; the presumed elimination pathway is primarily the fecal route, with limited hepatic metabolism by cytochrome P450 3A4, UDP-glucuronosyltransferase, and glutathione S-transferase [14]. In elderly individuals and patients with renal or hepatic impairment, physiological changes associated with aging and organ dysfunction might lead to altered protein binding of medications. For drugs that are highly protein-bound, such as selinexor, changes in protein-binding characteristics possibly lead to substantial variability in unbound drug concentrations. Considering that free drug concentrations account for pharmacological and toxicological activities, the need for selinexor dosing adjustment should be considered based on changes in unbound concentrations [2,15,16]. However, previously published studies evaluated the pharmacokinetics of selinexor based on total concentrations without measuring unbound concentrations [10,14]. Thus, there is a paucity of data to suggest dosing adjustment of selinexor based on unbound drug concentrations, particularly in specific patient populations with potentially altered protein binding.

For pharmacokinetic assessment of selinexor to individualize therapy based on unbound concentrations, a fully validated, high-throughput bioanalytical assay is warranted to accurately and precisely measure unbound selinexor concentrations in human plasma [14]. However, previously published analytical methods were developed to determine total selinexor concentrations in plasma samples from human or animals [17,18,19,20,21]. None of the currently available bioanalytical assays were validated to quantify unbound selinexor concentrations in plasma. Moreover, a previously developed method to quantitate total selinexor concentrations in human plasma requires plasma samples of ≥100 μL, which might be relatively large, particularly for elderly or renally impaired patients with multiple myeloma, who often experience myelosuppression or limited vascular access [21]. A thoroughly validated and highly efficient bioanalytical method requiring a minimal sample volume (<100 μL) would enables rapid, accurate, and precise measurement of unbound selinexor levels across numerous human plasma samples; ultimately, this new analytical method may facilitate clinical pharmacokinetic studies of selinexor in diverse patient populations, including patients with severe renal impairment, where the relationship between selinexor exposures, particularly unbound exposures, and pharmacodynamic responses has not been clearly established [10,13].

Hence, the main purpose of this study was to establish an efficient, accurate, and robust bioanalytical assay for quantification of total and unbound selinexor in human plasma. Our newly developed and fully validated analytical method was applied to an ongoing clinical pharmacokinetic study in patients with multiple myeloma to measure total and unbound concentrations of selinexor in serial human plasma samples, suggesting clinical validity and feasibility. In summary, this study introduces a novel LC-MS/MS assay that, for the first time, enables simultaneous quantification of total and unbound selinexor in human plasma. This method uniquely requires only a small plasma volume and offers a rapid runtime, representing a significant advancement in the analytical procedure, especially for pharmacokinetic studies in specific populations (e.g., renally impaired multiple myeloma patients) requiring a high-throughput bioanalytical assay.

## 2. Materials and Methods

### 2.1. Chemicals and Reagents

Selinexor (purity: >98.0%) was procured from Adooq Bioscience and stored at −20 °C. Sitagliptin (internal standard [IS], 97.1% purity) was provided by Daewon Pharmaceutical Co. and kept at room temperature. All solvents and reagents were of HPLC analytical grade, unless otherwise noted. Blank human plasma treated with K2-EDTA was purchased from BioChemed (Winchester, VA, USA), stored at −80 °C (±10 °C), and used for calibration standards and quality-control (QC) samples. Stock solutions of selinexor (1 mg/mL) and IS (sitagliptin, 1 mg/mL) were prepared in 100% methanol, with working solutions at various concentrations prepared by diluting stocks in 50% methanol (*v*/*v*). All solutions were stored at −20 °C. The QC samples were separately prepared, relative to the working solutions, at 15, 750, and 1600 ng/mL for total selinexor and at 0.15, 3, and 16 ng/mL for unbound selinexor. The calibration standard samples were generated by spiking blank human plasma with working solutions to achieve total selinexor concentrations of 5 (lower limit of quantification, LLOQ), 20, 50, 200, 500, 1000, and 2000 (upper limit of quantification, ULOQ) ng/mL and at 0.05 (LLOQ), 0.25, 1, 2, 5, 10, and 20 (ULOQ) ng/mL for unbound selinexor.

### 2.2. Instrumentation and Analytical Conditions

Chromatographic separation was performed using an Agilent 1200 Series HPLC system (Agilent Technologies, Santa Clara, CA, USA), equipped with a Kinetex C18 column (100 × 2.0 mm, 2.6 µm, Phenomenex, Torrance, CA, USA) maintained at 40 °C. Among the various mobile phases tested (isocratic or gradient, using combinations of 0.1% formic acid, methanol, and acetonitrile), the final mobile phase chosen was 0.1% formic acid in water and 100% methanol at a 12:88 (*v*/*v*) ratio and a 0.2 mL/min flow, with a total run time of 2.5 min. The sample injection volume was 5 μL (total selinexor) or 7 μL (unbound). The autosampler was set at 10 °C.

**Figure 1:**
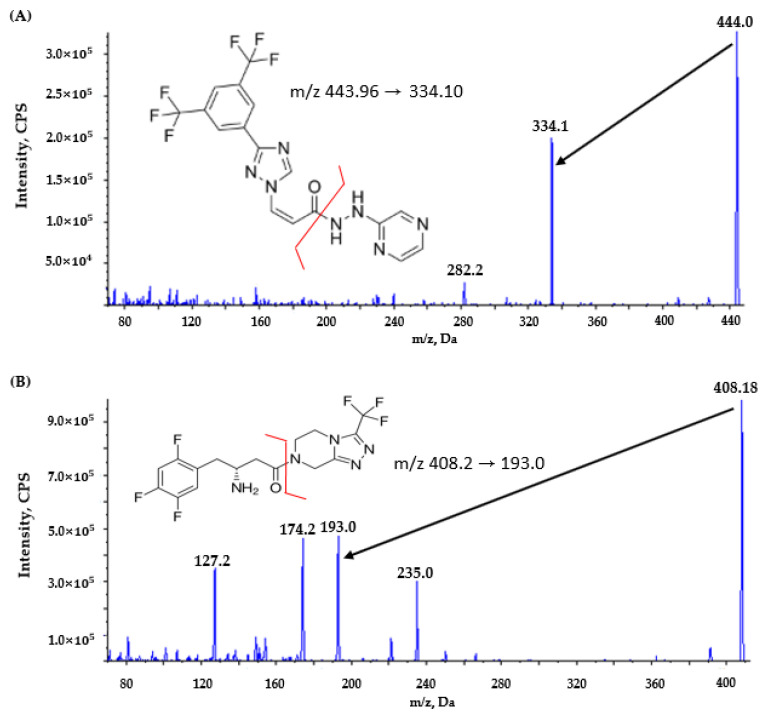
Product ion mass spectra and fragmentation patterns of (**A**) selinexor and (**B**) sitagliptin (internal standard, IS). Black arrows represent the *m*/*z* transition of the primary product ion from 444 to 334 in (**A**) and from 408 to 193 in (**B**) for quantitative analysis in the multiple-reaction-monitoring (MRM) mode. Red lines show the specific fragmentation sites resulting in the formation of product ions for (**A**) selinexor and (**B**) sitagliptin.

Mass spectrometric detection was conducted using an AB SCIEX API 4000 triple-quadrupole mass spectrometer (Framingham, MA, USA) in the positive electrospray ionization mode (ESI+). Instrumental source parameters included the following: ion spray voltage of 5500 V, source temperature of 550 °C, curtain gas at a pressure of 20 psi, and collision gas at a pressure of 7 psi. Selinexor and IS (sitagliptin) were detected using the multiple-reaction-monitoring (MRM) mode, with the transitions set at *m*/*z* 444 → 334 for selinexor and *m*/*z* 408 → 193 for sitagliptin (IS) (Figure 1). The optimized mass spectrometry parameters for selinexor (*m*/*z* 444 → 334) and sitagliptin (IS, *m*/*z* 408 → 193) are presented in Table 1. These settings provided the optimal quantitative sensitivity despite the visibly noticeable response intensity of the parent ions in the product’s ion spectra. Data acquisition and processing were performed using Analyst^®^ software version 1.6.2.

### 2.3. Sample Preparation

To quantify the total selinexor in plasma, calibration and QC samples (50 µL each) were freshly prepared with 45 µL of human plasma spiked with 5 µL of selinexor working solutions of various concentrations. Afterward, 20 µL of IS working solution (sitagliptin, 2500 ng/mL) was combined with the QC samples and calibration standards. Liquid–liquid extraction (LLE) was performed for each standard sample by adding 1 mL of methyl tertiary-butyl ether (MTBE), as an extraction solvent, to the standard samples mixed with IS, followed by vortexing for 10 min [22]. Notably, the LLE procedure effectively extracts both unbound and bound fractions of selinexor from plasma proteins, ensuring that the total (i.e., protein-bound plus unbound) drug is recovered without a separate protein precipitation step. After centrifuging the samples at 20,000× *g* for 10 min at 4 °C, the clear supernatant from each tube was carefully transferred to a fresh tube. The solvent was evaporated under nitrogen gas at 40 °C, and the residue was reconstituted with 500 µL of 90% methanol. The reconstituted solution was centrifuged at 20,000× *g* for 10 min. Subsequently, 50 µL supernatant of the reconstituted solution was taken and diluted with 450 µL of 90% methanol and then 5 µL of the diluted solution was injected into the LC-MS/MS system.

For the unbound selinexor measurement, plasma ultrafiltration was performed using Amicon^®^ Ultra-0.5 Centrifugal Filter Devices (50 kDa molecular weight cutoff (MWCO) and 9.4 mm filter membrane diameter, Sigma-Aldrich, Steinheim, Germany) [15]. Considering the reversible protein binding of selinexor in plasma, ultrafiltration was deemed to be an appropriate method to quantify the unbound form, ensuring the dynamic equilibrium between the bound and unbound states [23,24]. Ultrafiltration was chosen to obtain the unbound fraction while preserving the protein-binding equilibria, thus avoiding the need for protein denaturation before the LLE. A 300 µL aliquot of plasma was centrifuged to obtain plasma ultrafiltrate by removing the bound form of selinexor [23,25,26]. A 270 µL portion of plasma ultrafiltrate was spiked with 30 µL of selinexor working solutions and 20 µL of IS (sitagliptin, 150 ng/mL). The extraction procedure was the same as that for the total selinexor using LLE with 1.5 mL of MTBE. The extracted solution was dried under nitrogen gas at 40 °C. The residue was reconstituted with 500 µL of 90% methanol. The reconstituted solution was centrifuged at 20,000× *g* for 10 min, and a 7 µL aliquot of the reconstituted solution was injected into the LC-MS/MS system.

### 2.4. Method Validation

The validation process was conducted in accordance with bioanalytical method validation guidelines issued by both the U.S. Food and Drug Administration (FDA) and the Ministry of Food and Drug Safety (MFDS) of Korea [27,28]. These guidelines include assessments of the selectivity, linearity, precision, accuracy, recovery, matrix effect, and stability. The selectivity was evaluated by analyzing six different blank human plasma samples and pooled samples to ensure no interfering peaks at the retention times of selinexor and IS. The acceptance criteria for selectivity were defined as the presence of additional peaks, with ≤20% of the peak area for selinexor at the LLOQ and ≤5% for IS. For linearity, calibration curves were constructed separately for total and unbound selinexor over a concentration range from 5 (LLOQ) to 2000 (ULOQ) ng/mL and from 0.05 (LLOQ) to 20 (ULOQ) ng/mL, respectively. Linearity (y = ax + b) was assessed using a weighted linear regression model to establish the relationship between total or unbound selinexor concentrations (x) and the peak area ratios of the analyte to IS (y) using 1/x^2^ as a weighting factor. The LLOQ was defined as the minimum measurable concentration demonstrating a signal-to-noise ratio (S/N) equal to or greater than 10. The S/N was calculated by dividing the analyte peak height, as the signal, by the baseline height over a region approximately 3× the signal peak width, as the noise, using Analyst^®^ software.

Precision and accuracy were determined using five replicates at the following four QC standard concentrations: 5 [LLOQ], 15, 750, and 1600 ng/mL for total selinexor and 0.05 (LLOQ), 0.15, 3, and 16 ng/mL for unbound selinexor. Intra-day precision and accuracy were determined by analyzing five replicates within a single day, whereas inter-day precision and accuracy were evaluated by analyzing five replicates per day across three consecutive days. Between-run precision and accuracy were assessed over three days with five replicates each day. Accuracy is expressed as the percentage of the mean predicted concentration relative to the nominal concentration. Precision was calculated as the percent coefficient of variation (%CV). Precision and accuracy were considered acceptable with a %CV within ±15% and deviations from the nominal concentrations within ±15%, respectively, except for the LLOQ, for which the acceptance criteria were within ±20%.

The extraction recovery and matrix effect were investigated using six replicates at the following three QC concentrations: 15 (low), 750 (medium), and 1600 (high) ng/mL for total selinexor and 0.15 (low), 3 (medium), and 16 (high) ng/mL for unbound selinexor. Recovery was determined by comparing the peak areas of the post-extraction spiked samples with the pre-extraction spiked samples at the three QC concentrations. Matrix effects were evaluated by calculating the ratios of the peak areas of the post-extraction spiked samples to those of the pure solutions. Acceptable criteria for the matrix effects and extraction recovery were defined as the %CVs for the measured concentrations at each QC concentration within ±15%. Carryover was assessed by analyzing a double-blank sample after the highest-concentration calibration standard (ULOQ). Carryover was deemed to be acceptable if any peaks observed were ≤20% of the peak response at the LLOQ for selinexor and ≤5% of the IS peak response.

The stability of selinexor in the stock and working solutions was examined in three replicates at low-QC (15 ng/mL for total and 0.15 ng/mL for unbound) and high-QC (1600 ng/mL for total and 16 ng/mL for unbound) concentrations at room temperature for 3 h for the stock solutions or 7 h for the working solutions. The stability of IS in the stock and working solutions was assessed at a concentration of 2500 ng/mL for the total form and 150 ng/mL for the free form, with three replicates under the same conditions as selinexor. The stability of selinexor in plasma was tested under the following conditions: (a) freeze–thaw stability after three cycles at −70 °C; (b) short-term stability at room temperature, 4 °C, and −70 °C for 7 h; and (c) extracted sample stability in the autosampler at 10 °C for 52 h. For the long-term stability assessment, the tested storage period was 370 days, which was the longest time that elapsed between the first sample collection date and the last sample analysis date. The acceptance criterion for stability was within a ±15% deviation of the mean prediction value from its nominal concentration.

### 2.5. Application of the Method to an Ongoing Pharmacokinetic Study

The validated LC–MS/MS assay was used to quantify the total and unbound selinexor in plasma samples from three patients with multiple myeloma receiving 80 mg selinexor on days 1 and 3 of each week [29,30]. All patients received 80 mg selinexor orally on days 1 and 3 of each weekly cycle of anticancer therapy. Patients included in this study were renally impaired, including those with a glomerular filtration rate below the 20 mL/min/1.73 m^2^ estimated by the CKD-EPI creatinine equation [31,32,33]. On the first day of the selinexor therapy, blood samples were collected in K2-EDTA tubes pre-dose and, subsequently, at scheduled intervals of 1, 2, 4, 8, 12, and 24 h after administration. Plasma samples were isolated through centrifugation at 2691× *g* for 10 min and kept at −70 °C until analysis. Ultrafiltration (Amicon^®^, Sigma-Aldrich, Steinheim, Germany) was used to separate the free fraction before the LLE for the unbound assay. This study was approved by the Institutional Review Board (IRB) of St. Mary’s Hospital in Seoul, South Korea (IRB no.: KC23MISS0087).

## 3. Results and Discussion

### 3.1. Development of LC-MS/MS Method

**Figure 2:**
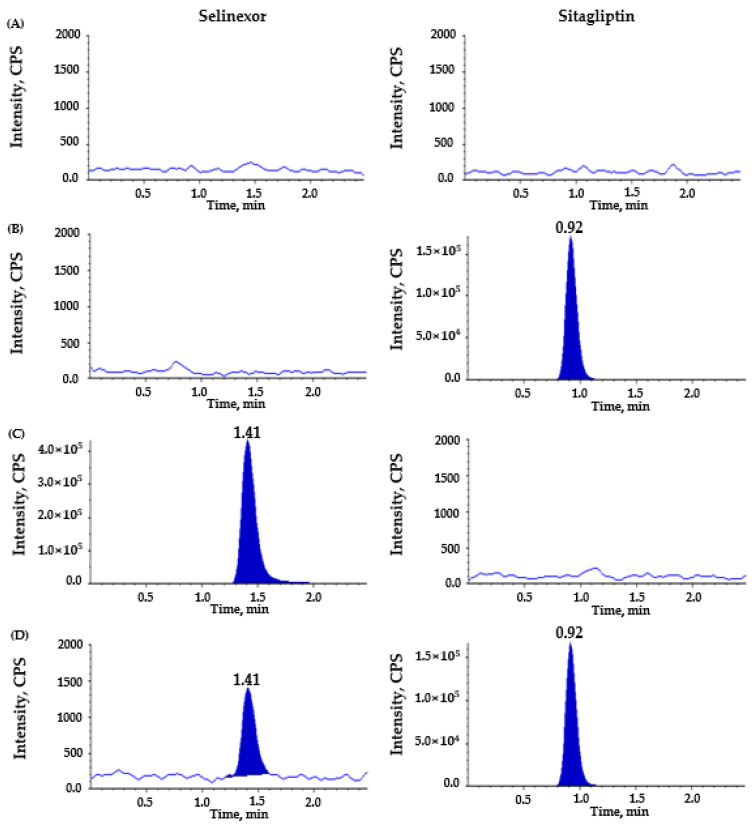
Representative chromatograms for total selinexor in human plasma: (**A**) double blank; (**B**) blank spiked with sitagliptin, as the internal standard (IS, 2500 ng/mL); (**C**) blank spiked with selinexor (upper limit of quantification, 2000 ng/mL); (**D**) blank spiked with selinexor (lower limit of quantification, 5 ng/mL) and sitagliptin (IS, 2500 ng/mL). Panels on the left side are for selinexor, and those on the right side are for IS.

**Figure 3:**
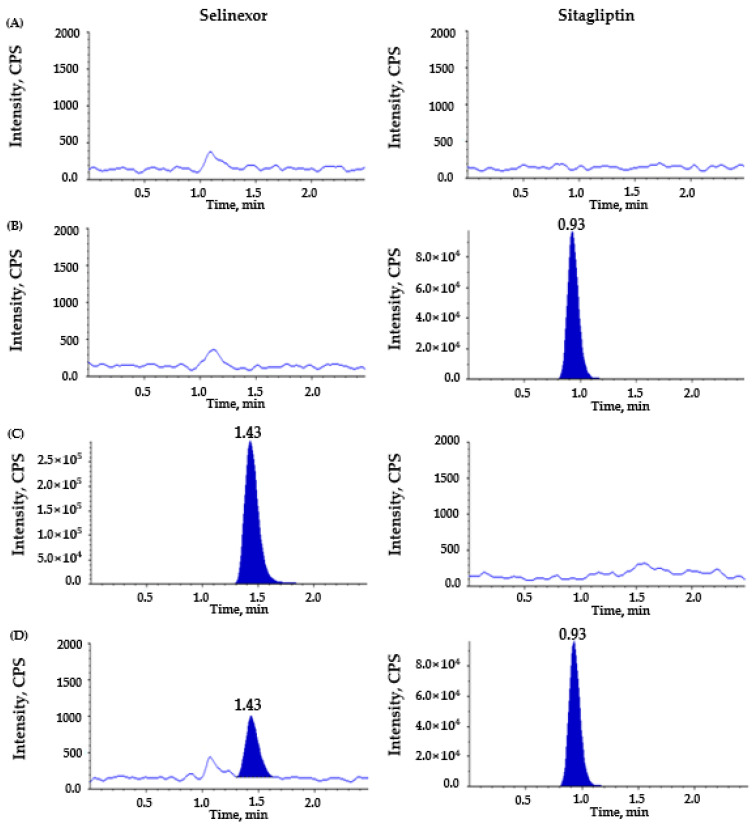
Representative chromatograms for unbound selinexor in post-ultrafiltration plasma: (**A**) double blank; (**B**) blank spiked with sitagliptin, as an internal standard (IS, 150 ng/mL); (**C**) blank spiked with selinexor (upper limit of quantification, 20 ng/mL); (**D**) blank spiked with selinexor (lower limit of quantification, 0.05 ng/mL) and sitagliptin (IS, 150 ng/mL). Left-side panels are for selinexor and the right-side are for IS.

To establish an efficient, accurate, and reproducible LC–MS/MS approach for total and unbound selinexor, multiple chromatographic and mass spectrometric settings were tested. Several reverse-phased columns were evaluated to optimize the chromatographic separation of selinexor and sitagliptin (IS), including Kinetex^®^ C18 (100 × 2.0 mm, 2.6 µm, Phenomenex, Torrance, CA USA), Luna^®^ C18 (50 × 2.1 mm, 3.0 µm, Phenomenex, Torrance, CA, USA), and YMC-Pack C8 column (50 × 2.1 mm, 3.0 µm, YMC, Kyoto, Japan). Among the columns tested, the Kinetex^®^ C18 column was ultimately selected due to its superior peak shape, minimal tailing, excellent reproducibility, and selectivity for selinexor compared with the Luna^®^ C18 and the YMC-Pack C8 columns. Peak symmetry was quantitatively assessed using the tailing factor for the Kinetex^®^ C18 column, which is consistently estimated to be less than 1.2, indicating an excellent chromatographic performance and reproducible retention (~1.4 min for selinexor) (Figure 2 and Figure 3, Figure S1). Other columns produced either peak broadening or interfering matrix peaks near the selinexor retention time.

In addition, the optimized mobile phase consisting of 0.1% formic acid and 100% methanol (12:88, *v*/*v*) in the isocratic mode at a flow rate of 0.2 mL/min produced symmetrical peak shapes with short and reproducible retention times of 1.4 and 0.9 min for selinexor and IS (Figure 2 and Figure 3), respectively. This mobile phase composition provided a better chromatographic performance compared with previously evaluated conditions, including a gradient elution with formic acid and acetonitrile [20]. Specifically, in our repeated experiments, acetonitrile-based isocratic or gradient mobile phases typically eluted selinexor too early (often near the solvent front), increased the matrix background, and resulted in peak broadening or baseline drift (Figure S2); therefore, they were considered to hinder precise quantification. In contrast, the selected 12:88 (*v*/*v*) ratio of formic acid in a water and methanol mixture effectively balanced the retention, peak sharpness, and baseline stability for both the total plasma and ultrafiltrate sample analyses. Compared with previous studies [17,18,19,20,21], the total run time was relatively short at 2.5 min, suggesting an efficient method for a high-throughput analytical tool. Even with this short isocratic run time, selinexor and IS were well-resolved from any endogenous peaks, as evidenced by the clean baseline in the double-blank plasma chromatograms (Figure 2A and Figure 3A).

The mass spectrometry conditions were optimized in ESI+ at 550 °C. We noted strong protonated molecular ions at *m*/*z* 444 (selinexor) and *m*/*z* 408 (sitagliptin), which fragmented to *m*/*z* 334 and *m*/*z* 193, respectively. Tuning gas flows, including curtain, collision, and ionized gas, minimized the noise and maximized the analyte signal intensity.

### 3.2. Sample Preparation

A simple LLE procedure with MTBE achieved adequate chromatographic separation for both the total and unbound selinexor assays. Although a previous study used the protein precipitation (PP) method with acetonitrile for the sample preparation, unbound selinexor concentrations were not measured in that particular study. Considering a very low fraction of unbound selinexor (<5% of total), LLE was preferred to PP for quantifying a very low unbound selinexor concentration of 0.05 ng/mL.

In contrast to a previous study using 100 μL of human plasma for selinexor quantification [20], this assay used only 50 μL for the total selinexor measurements, making it more suitable for the high-throughput analysis required for pharmacokinetic evaluation of selinexor in real-world practice, as well as research purposes. For the unbound measurement, we employed ultrafiltration (Amicon^®^ Ultra-0.5 devices, Sigma-Aldrich, Steinheim, Germany) to isolate the free fraction [34] and then performed the same LLE process.

### 3.3. Validation of the LC-MS/MS Method

To our knowledge, this was the first study to develop and fully validate a bioanalytical assay capable of measuring both total and free selinexor in human plasma. Our newly developed analytical approach showed comparable selectivity, precision, accuracy, and stability to a previously published method [20], with negligible carryover, sufficient recovery, and no substantial interferences. The results below confirm that our approach meets all relevant guidelines [27,28]. Detailed results of the validation test for our newly developed bioanalytical assay are provided below.

#### 3.3.1. Selectivity and Sensitivity

The representative chromatograms of the double-blank sample, sitagliptin (IS)-spiked blank sample, selinexor-spiked blank sample, and blank sample spiked with both selinexor and IS are shown in Figure 2 for human plasma and in Figure 3 for plasma ultrafiltrate. Overall, no substantial interfering peaks were detected around the retention time of selinexor (total: 1.41 min; unbound: 1.43 min) and IS (total: 0.92 min; unbound: 0.93 min), indicating sufficient selectivity and specificity by our newly developed analytical method to measure total and unbound selinexor concentrations in plasma. The LLOQ was 5 ng/mL for total selinexor and 0.05 ng/mL for unbound selinexor, each with an S/N ratio of 10 or higher, suggesting excellent sensitivity even at a very low concentration of unbound selinexor due to high protein binding in human plasma [3,13,14].

#### 3.3.2. Linearity

Linearity was assessed by constructing calibration curves over a concentration range of 5–2000 ng/mL for total selinexor and 0.05–20 ng/mL for unbound selinexor in human plasma. The estimated coefficients of determination and correlation coefficients were ≥0.998 and ≥0.9992, respectively, for the total selinexor calibration curves using linear regression with 1/x^2^ as a weighting factor. For unbound selinexor, the calibration curves showed coefficients of determination ≥0.995 and correlation coefficients ≥0.9975. Representative linear equations with the mean ± standard deviation (SD) for the slope and the y-intercept were as follows:

total selinexor: y = 0.00180 (±0.00007)x − 0.00233 (±0.00095)

unbound selinexor: y = 0.20425 (±0.00512)x + 0.00070 (±0.00034)

#### 3.3.3. Precision and Accuracy

Table 2 summarizes the within- and between-run precision and accuracy data for the total and unbound selinexor in plasma, expressed as the %CV and % ratio of the average predicted concentration to the nominal concentration. For determination of the total selinexor, the intra- and inter-run precisions were ≤6.00% and ≤8.67%, respectively; the within- and between-run accuracies ranged from 95.84% to 101.75% and from 95.77% to 104.13%, respectively. Regarding unbound selinexor, the within- and between-day precisions were ≤6.48% and ≤10.35%, respectively; the intra- and inter-day accuracies ranged from 92.80% to 107.24% and from 92.46% to 105.61%, respectively. Overall, our newly developed bioanalytical method satisfied the pre-defined precision and accuracy acceptance criteria according to the bioanalytical assay validation guidelines established by the FDA and the MFDS, suggesting a reliable and robust analytical method to quantitate the total and unbound selinexor in human plasma [27,28].

#### 3.3.4. Recovery, Matrix Effect, and Carryover

Following the LLE for the quantification of the total selinexor concentrations in plasma, the mean extraction recoveries at the three QC levels ranged from 88.26% to 94.86% (Table 3). For unbound selinexor, following the ultrafiltration and subsequent LLE, the recoveries (88.87–92.71%) were comparable to those of total selinexor (Table 3). The %CVs for recovery were ≤3.55% (total) and ≤7.23% (unbound), fulfilling the criteria set by both the FDA’s and the MFDS’s guidelines [27,28]. These outcomes indicate that our sample preparation steps were consistent and sufficiently robust for quantifying both total and free selinexor.

The matrix effect estimates for total selinexor were between 103.71% and 120.16%, whereas the values for unbound selinexor fell between 101.18% and 103.65% (Table 3). For IS, the mean matrix effects were 102.31% and 99.99% for the total and unbound forms, respectively (Table 3). Despite numerically apparent ion-enhancement effects, the corresponding CVs remained at or below 6.93% (total) and 5.82% (unbound). Moreover, the chromatograms revealed no additional peaks interfering with selinexor or IS at their retention times. Accordingly, our newly developed bioanalytical assay satisfied the relevant regulatory guidelines [27,28], demonstrating minimal impact by the matrix components.

Carryover was evaluated by injecting a double-blank sample immediately after the ULOQ calibration standard. No selinexor or IS peaks were detected in those blank injections, implying negligible carryover. Overall, this indicates that our assay can reliably process large numbers of clinical samples in sequence without contamination.

#### 3.3.5. Stability

We assessed the stability of selinexor and IS under various conditions, encompassing the stock and working solutions, as well as the plasma samples. For the total selinexor in the stock solutions at 15 and 1600 ng/mL, the mean ± SD of the stability estimates were 100.54 ± 2.66% and 100.40 ± 2.70%, respectively. The IS (2500 ng/mL) displayed a stability of 100.89 ± 2.29%. In the working solutions, selinexor remained at 97.05 ± 3.15% and 99.23 ± 1.03% for the same QC levels, and the IS was 97.10 ± 0.66%. When evaluating the unbound selinexor at the stock concentrations of 0.15 and 16 ng/mL, we observed stability values of 89.39 ± 7.81% and 96.29 ± 3.30%, respectively, whereas the IS (150 ng/mL) showed 100.43 ± 4.21%. In the working solutions, the unbound selinexor’s stability was measured at 101.71 ± 5.49% and 99.19 ± 3.83% for the same two QC levels, while the IS’s stability was 100.81 ± 0.33%.

The plasma sample stability was verified under freeze–thaw, short-term, and autosampler conditions, as well as long-term storage. Across these tests, the total selinexor concentrations remained between 94.18% and 110.69% of the nominal, whereas the unbound selinexor ranged from 88.00% to 102.99% (Table 4). Overall, our newly developed bioanalytical assay satisfied the acceptance criteria for stability, suggesting a lack of substantial degradation of the analytes, with sufficient stability and integrity by the analytes in the plasma samples, as well as in the stock and working solutions, throughout the laboratory and analytical procedures.

### 3.4. Method Application and Future Directions

**Figure 4:**
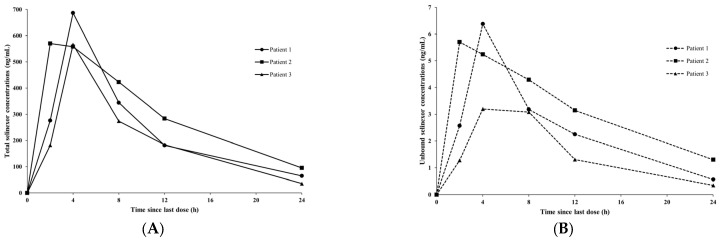
Observed (**A**) total and (**B**) unbound selinexor concentrations in plasma at multiple time points from three patients with multiple myeloma (Patients 1, 2, and 3) orally receiving 80 mg selinexor.

Our newly established LC–MS/MS method was applied to measure the total and unbound selinexor in 18 plasma samples obtained from three patients with multiple myeloma (6 samples from each patient). Each patient received an 80 mg oral dose of selinexor, and sampling was performed at multiple time points. Figure 4 shows the plasma concentration–time profiles of the total and unbound selinexor for the three patients. Based on our measurements, the median unbound fraction was approximately 0.94% (range: 0.56–1.36%). In all subjects, both the total and unbound selinexor reached peak concentrations within four hours post-dose, and the observed values fell within the validated calibration ranges of our assay (35.41–687.08 ng/mL for total selinexor and 0.35–6.38 ng/mL for the unbound form).

The maximum total selinexor concentration measured in this study (603.76 ± 72.26 ng/mL) is suggested to be consistent with earlier reports in patients with hematologic malignancies, where the reported mean ± SD maximum plasma concentration (Cmax) was 680 ± 124 ng/mL, according to the prescribing information [8,10,14]. In contrast, one previous study in Chinese patients with multiple myeloma reported relatively lower Cmax values of selinexor (265.68 ± 206.9 ng/mL) [20]. The difference in the measured maximum plasma concentrations of the total selinexor in this study might be accounted for by the administration of a lower selinexor dose of 60 mg, where Cmax was measured at 3 h following selinexor administration. Additionally, the patients in our clinical sample set had renal impairment. Impaired kidney function, while not the primary elimination route for selinexor, could lead to physiological changes (e.g., altered protein binding or metabolism), potentially slowing clearance.

Overall, our newly developed and fully validated bioanalytical method based on LC-MS/MS is suggested to be simple, efficient, and robust with a short run time and a relatively small plasma volume required for analysis, indicating its appropriateness as a high-throughput assay to quantitate total and unbound selinexor in a large number of clinical plasma samples for routine therapeutic drug-monitoring practice, as well as for research purposes. The measured total and unbound selinexor concentrations in the clinical samples from three patients in our ongoing clinical pharmacokinetic study further support the validity of our newly developed LC-MS/MS assay for total and unbound selinexor in human plasma. In addition to its application in clinical research, our bioanalytical assay may be useful in clinical practice to monitor total and unbound selinexor concentrations in oncology patients, particularly in specific populations such as patients with severe kidney dysfunction, where the variability in protein binding may be high, potentially leading to substantially altered drug exposure. Considering the high protein-binding fraction of selinexor with large variability, accurate measurement of unbound selinexor concentrations with a small plasma volume is imperative for safe and effective drug therapy in clinical practice, especially for such patients where protein binding is potentially altered [35], as in renally impaired patients. Therefore, our current study might contribute to enhancing our understanding regarding selinexor pharmacokinetics, particularly in terms of protein binding, as well as individualizing selinexor dosing through precision medicine approaches, particularly in specific populations such as renally impaired patients, who are frequently encountered among individuals with multiple myeloma. By enabling the reliable measurement of unbound selinexor concentrations, our assay provides a useful tool for clinicians, as well as researchers, to better understand patient-specific pharmacokinetics of selinexor, especially in renally impaired or elderly patients with potentially altered protein binding. For instance, if the unbound selinexor concentrations measured by our analytical method are found to be higher than anticipated for the total concentrations due to reduced protein binding, dose reduction should be considered based on the results of our assay to avoid toxicity. Similarly, if a patient has unusually low systemic exposures of unbound selinexor compared with total drug exposures, doses or dosing frequency might be increased to achieve adequate unbound systemic exposures for efficacy. In essence, our method contributes to forming the groundwork for the precision dosing of selinexor by tailoring therapy based on unbound drug exposure measurement in special populations.

## 4. Conclusions

We developed a fully validated LC–MS/MS assay to simultaneously quantify total and unbound selinexor in human plasma. Unlike previous studies exclusively measuring total selinexor concentrations, our approach also captures the free fraction using ultrafiltration followed by LLE. With a relatively short run time of 2.5 min using a smaller plasma volume of 50 μL, our LC-MS/MS bioanalytical method might be used as an efficient, high-throughput assay to quantitate total and unbound selinexor in a large number of human plasma samples, making it pertinent for monitoring the treatment outcomes of selinexor in clinical practice, as well as optimizing dosing regimens of selinexor in clinical research, particularly for specific populations such as renally impaired patients.

## Figures and Tables

**Table 1:** Mass spectrometer conditions for selinexor and sitagliptin (IS).

Compound	DP (V)	EP (V)	CE (V)	CXP (V)	RT (min)
Selinexor	78	11	30	16	1.4
Sitagliptin	91	9	30	40	0.9

DP: declustering potential; EP: entrance potential; CE: collision energy; CXP: cell exit potential; RT: retention time.

**Table 2:** Intra- and inter-run precision and accuracy for quantitation of the total and unbound selinexor in human plasma.

Nominal Concentration (ng/mL)	Intra-Run (*n* = 5 Sample Replicates)			Inter-Run (*n* = 3 Runs with 5 Replicates/Run)		
	Predicted Concentration (Mean ± SD) (ng/mL)	Precision (CV, %) ^a^	Accuracy (%) ^b^	Predicted Concentration (Mean ± SD) (ng/mL)	Precision (CV, %) ^a^	Accuracy (%) ^b^
*Total selinexor*						
5	5.07 ± 0.30	6.00	101.42	5.21 ± 0.45	8.67	104.13
15	14.39 ± 0.44	3.08	95.97	14.37 ± 0.68	4.75	95.77
750	763.14 ± 13.80	1.81	101.75	755.33 ± 17.41	2.31	100.71
1600	1533.47 ± 14.19	0.93	95.84	1539.65 ± 27.66	1.80	96.23
*Unbound selinexor*						
0.05	0.053 ± 0.003	6.48	105.60	0.050 ± 0.005	10.35	99.07
0.15	0.139 ± 0.001	0.60	92.80	0.14 ± 0.01	4.94	92.46
3	2.90 ± 0.06	2.06	96.69	2.91 ± 0.09	3.17	97.06
16	17.16 ± 0.28	1.63	107.24	16.90 ± 0.51	2.99	105.61

^a^ Precision (CV, %) = (standard deviation of the predicted concentrations/nominal concentration) × 100. ^b^ Accuracy (%) = (predicted concentration/nominal concentration) × 100. CV: coefficient of variation; SD: standard deviation.

**Table 3:** Extraction recovery and matrix effect of selinexor and sitagliptin (internal standard, IS) in human plasma using liquid–liquid extraction (*n* = 6, different blank plasma).

Nominal Concentration (ng/mL)	Recovery (%)		Matrix Effect (%)	
	Mean ± SD	%CV	Mean ± SD	%CV
Total selinexor				
15	88.26 ± 3.13	3.55	109.40 ± 7.58	6.93
750	94.86 ± 1.06	1.11	103.71 ± 2.09	2.02
1600	88.40 ± 2.28	2.58	120.16 ± 2.53	2.11
IS (2500)	88.53 ± 3.49	3.94	102.31 ± 2.08	2.03
Unbound selinexor				
0.15	88.87 ± 6.42	7.23	103.65 ± 6.03	5.82
3	92.15 ± 2.55	2.77	101.50 ± 1.78	1.76
16	92.71 ± 0.82	0.89	101.18 ± 1.76	1.74
IS (150)	75.26 ± 2.25	2.99	99.99 ± 2.13	2.13

CV: coefficient of variation; SD: standard deviation.

**Table 4:** Stability data (mean ± standard deviation, %) for total and unbound selinexor in human plasma.

Stability Condition	Analyte Nominal Concentration (ng/mL)					
	Total Selinexor			Unbound Selinexor		
	15	750	1600	0.15	3	16
Freeze–thaw, three cycles	97.98 ± 2.66	100.23 ± 1.67	95.25 ± 1.17	89.11 ± 1.88	96.54 ± 0.73	100.84 ± 0.90
Room temperature, 7 h	94.62 ± 3.42	100.50 ± 0.54	97.13 ± 2.04	93.56 ± 1.79	94.66 ± 8.07	99.25 ± 1.55
4 °C, 7 h	95.90 ± 2.29	99.27 ± 0.24	95.42 ± 0.44	88.00 ± 1.31	98.46 ± 8.07	98.09 ± 2.85
−70 °C, 7 h	96.70 ± 3.21	101.39 ± 0.77	95.44 ± 0.90	88.00 ± 2.27	97.59 ± 0.64	101.96 ± 3.16
Autosampler (10 °C), 30 h	94.18 ± 3.78	101.25 ± 1.47	95.03 ± 0.43	88.44 ± 3.56	94.69 ± 1.27	102.99 ± 1.71
Long-term storage (−70 °C), 370 days	110.69 ± 3.54	109.57 ± 2.70	103.08 ± 0.45	100.44 ± 6.65	89.20 ± 2.37	93.87 ± 7.44

## Data Availability

The data presented in this study may be available upon request.

## Supplementary Materials

The following supporting information can be downloaded at: https://www.mdpi.com/article/10.3390/pharmaceutics17070919/s1, Figure S1: Representative chromatograms for selinexor and sitagliptin (internal standard [IS]) in various chromatographic conditions using the YMC-Pack C8 column [(A) to (C)] and the Luna® C18 column [(D) to (F)]. (A), simultaneous injection of selinexor (5 ng/mL) and sitagliptin (2500 ng/mL) showed the fronting and asymmetrical peak shape. (B), injection of 100% methanol as a wash solvent resulted in a baseline drift where the signal dropped from a higher intensity to a lower intensity during data collection with an unknown peak at the selinexor peak retention time. (C) injection of sitagliptin without selinexor resulted in an unknown peak at the selinexor retention time. (D) sequential injection of Sample 41 (1000 ng/mL of selinexor) followed by Sample 42 showed a small amount of carryover in the wash vial of Sample 42. (E) addition of 50% methanol as a dilution solvent resulted in an unknown peak at the IS peak retention time. (F) sequential injection of Sample 44 (zero blank, sitagliptin only) followed by Sample 45 with both selinexor (5 ng/mL, LLOQ) and sitagliptin (2500 ng/mL) resulted in a greater peak height of selinexor in Sample 44 (zero blank) compared to that in Sample 45.; Figure S2: Representative chromatogram for selinexor in human plasma with the acetonitrile-based mobile phase showing elution of selinexor too early near the solvent front (k’ value <1).

## References

[1] Mo C.C., Yee A.J., Midha S., Hartley-Brown M.A., Nadeem O., O’Donnell E.K. (2023). Selinexor: Targeting a novel pathway in multiple myeloma. eJhaem.

[2] Peterson T.J., Orozco J., Buege M. (2020). Selinexor: A First-in-Class Nuclear Export Inhibitor for Management of Multiply Relapsed Multiple Myeloma. Ann. Pharmacother..

[3] Syed Y.Y. (2019). Selinexor: First Global Approval. Drugs.

[4] Kumar S.K., Callander N.S., Adekola K., Anderson L.D., Baljevic M., Baz R. (2023). Multiple Myeloma, Version 2.2024, NCCN Clinical Practice Guidelines in Oncology. J. Natl. Compr. Cancer Netw..

[5] White D., LeBlanc R., Venner C., Bahlis N.J., Lentzsch S., Gasparetto C.J. (2019). Safety and Efficacy of the Combination of Selinexor, Lenalidomide and Dexamethasone (SRd) in Patients with Relapsed/Refractory Multiple Myeloma (RRMM). Clin. Lymphoma Myeloma Leuk..

[6] White D., LeBlanc R., Baljevic M., Bahlis N., Lentzsch S., Venner C. (2020). Selinexor, Lenalidomide and Dexamethasone (SRd) for Patients with Relapsed/Refractory and Newly Diagnosed Multiple Myeloma. Blood.

[7] Mousavi S.E., Ilaghi M., Aslani A., Yekta Z., Nejadghaderi S.A. (2023). A population-based study on incidence trends of myeloma in the United States over 2000–2020. Sci. Rep..

[8] U.S. Food and Drug Administration (2019). XPOVIO (Selinexor) Tablets Prescribing Information. https://www.accessdata.fda.gov/drugsatfda_docs/label/2019/212306s000lbl.pdf.

[9] Huang Q., Zhao R., Xu L., Hao X., Tao S. (2024). Treatment of multiple myeloma with selinexor: A review. Ther. Adv. Hematol..

[10] Xu H., Li H., Wada R., Bader J.C., Tang S., Shah J. (2021). Selinexor population pharmacokinetic and exposure-response analyses to support dose optimization in patients with diffuse large B-cell lymphoma. Cancer Chemother. Pharmacol..

[11] Nooka A.K., Costa L.J., Gasparetto C.J., Richardson P.G., Siegel D.S., Chari A. (2022). Guidance for Use and Dosing of Selinexor in Multiple Myeloma in 2021: Consensus From International Myeloma Foundation Expert Roundtable. Clin. Lymphoma Myeloma Leuk..

[12] Dimopoulos M.A., Terpos E., Chanan-Khan A., Leung N., Ludwig H., Jagannath S. (2010). Renal impairment in patients with multiple myeloma: A consensus statement on behalf of the International Myeloma Working Group. J. Clin. Oncol..

[13] Martino E.A., Vigna E., Bruzzese A., Labanca C., Mendicino F., Lucia E. (2024). Selinexor in multiple myeloma. Expert Opin. Pharmacother..

[14] Bader J.C., Abdul Razak A.R., Shacham S., Xu H. (2021). Pharmacokinetics of Selinexor: The First-in-Class Selective Inhibitor of Nuclear Export. Clin. Pharmacokinet..

[15] Wright J.D., Boudinot F.D., Ujhelyi M.R. (1996). Measurement and analysis of unbound drug concentrations. Clin. Pharmacokinet..

[16] Bozic B., Rutner J., Zheng C., Ruckser R., Selimi F., Racz K. (2021). Advances in the Treatment of Relapsed and Refractory Multiple Myeloma in Patients with Renal Insufficiency: Novel Agents, Immunotherapies and Beyond. Cancers.

[17] Li S., Zhang Y., Cheng Q., Xin J., Dong Z., Qiu X. (2020). UPLC-MS/MS Measurement of the Effect of Isavuconazole, Itraconazole and Fluconazole on the Pharmacokinetics of Selinexor in Rats. Infect. Drug Resist..

[18] Zhou C., Wang H., Zhou C., Li C., Zhu M., Qiu X. (2021). Establishment and Verification of UPLC-MS/MS Technique for Pharmacokinetic Drug-Drug Interactions of Selinexor with Posaconazole in Rats. Drug Des. Dev. Ther..

[19] Sauter M., Foerster K.I., Benzel J., Pfister S., Pajtler K.W., Haefeli W.E. (2021). Bioanalysis of selinexor in mouse plasma micro-samples utilizing UPLC-MS/MS. J. Chromatogr. B.

[20] Yan X., He X., Yang X., Zhao Q., Lou Y. (2024). The development and validation of a liquid chromatography tandem mass spectrometry method for the quantification of selinexor and its application in Chinese multiple myeloma patients. Anal. Methods.

[21] Shih S.C., Visram A., Mian H. (2025). Treatment of elderly and frail myeloma patients. La Presse Méd..

[22] Yaswanth M., Sreekanth M., Valli S.S., Narayana D.V., Sreedhar V., Venkataramana K. (2022). Bioanalytical Method Development and Validation of Selinexor in Rat Plasma by Liquid Chromatography-Tandem Mass Spectrometry: Pharmaceutical Science-Pharmaceutical Analysis. Int. J. Life Sci. Pharma Res..

[23] Vuignier K., Schappler J., Veuthey J.L., Carrupt P., Martel S. (2010). Drug–protein binding: A critical review of analytical tools. Anal. Bioanal. Chem..

[24] Waters N.J., Obach R.S., Di L. (2014). Consideration of the unbound drug concentration in enzyme kinetics. Methods Mol. Biol..

[25] Jager N.G.L., Van Ewijk-Beneken Kolmer E., Aarnoutse R., Te Brake L.H.M. (2024). Influence of ultrafiltration conditions on the measurement of unbound drug concentrations: Flucloxacillin as an example. J. Antimicrob. Chemother..

[26] Thomas P. (2024). Calculating Unbound Drug Concentrations in Human Plasma, Serum, or Urine. J. Clin. Exp. Pharmacol..

[27] U.S. Department of Health and Human Services, Food and Drug Administration (2018). Bioanalytical Method Validation Guidance for Industry. https://www.fda.gov/media/70858/download.

[28] U.S. Food and Drug Administration (2022). M10 Guideline for Bioanalytical Method Validation and Study Sample Analysis. https://www.fda.gov/media/162903/download.

[29] U.S. Food and Drug Administration (2024). Pharmacokinetics in Patients with Impaired Renal Function—Study Design, Data Analysis, and Impact on Dosing and Labeling: Guidance for Industry. https://www.fda.gov/media/78573/download.

[30] Seoul St. Mary’s Hospital (2024). A Prospective Sponsor-Investigator Clinical Study for Dose-Finding of Selinexor in Relapsed and/or Refractory Multiple Myeloma (RRMM) Patients with Impaired Renal Function.

[31] Junk E., Tzivian L., Folkmane I., Folkmanis K., Jushinskis J., Strazda G. (2025). Major adverse cardiovascular events and hyperuricemia as an effect-modifying factor in kidney transplant recipients. World, J. Transplant..

[32] Delgado C., Baweja M., Crews D.C., Eneanya N.D., Gadegbeku C.A., Inker L.A. (2022). A Unifying Approach for GFR Estimation: Recommendations of the NKF-ASN Task Force on Reassessing the Inclusion of Race in Diagnosing Kidney Disease. Am. J. Kidney Dis..

[33] Kramer H.J., Jaar B.G., Choi M.J., Palevsky P.M., Vassalotti J.A., Rocco M.V. (2022). An Endorsement of the Removal of Race From GFR Estimation Equations: A Position Statement From the National Kidney Foundation Kidney Disease Outcomes Quality Initiative. Am. J. Kidney Dis..

[34] Toma C., Imre S., Vari C., Muntean D., Tero-Vescan A. (2021). Ultrafiltration Method for Plasma Protein Binding Studies and Its Limitations. Processes.

[35] Dasgupta A. (2002). Clinical utility of free drug monitoring. Clin. Chem. Lab. Med..

