# Supplementary material for: Quantification of Total and Unbound Selinexor Concentrations in Human Plasma by a Fully Validated Liquid Chromatography-Tandem Mass Spectrometry Method
Source: Pharmaceutics. 2025 Jul 16;17(7):919. doi: 10.3390/pharmaceutics17070919 (PMC12300842; doi:10.3390/pharmaceutics17070919)
Supplement: Supplementary file 1 [file pharmaceutics-17-00919-s001.zip › pharmaceutics-3678123-supplementary.pdf]

Supplementary Materials

Quantification of total and unbound selinexor concentrations in human plasma by a fully validated liquid chromatography-tandem mass spectrometry method

Suhyun Lee[1,2†], Seungwon Yang[3,4,†], Hyeonji Kim[1,3,4], Wang-Seob Shim[5], Eunseo Song[5], Seunghoon Han[6], Sung-Soo Park[7], Suein Choi[6], Sungpil Han[6], Sung Hwan Joo[1,3,4], Seok Jun Park[1,3,4], Beomjin Shin[1,3,4], Donghyun Kim[1,3,4], Hyeon Su Kim[1,3,4], Kyung-Tae Lee[1,5,8,i*] , Eun Kyoung Chung[1,3,4,5,8*]

[1] Department of Pharmacy, College of Pharmacy, Kyung Hee University, Seoul 02447, Republic of Korea

[2] Department of Pharmacy, College of Pharmacy, Woosuk University, Wanju 55338, Republic of Korea

[3] Department of Regulatory Science, Graduate School, Kyung Hee University, Seoul 02447, Republic of Korea

[4] Institute of Regulatory Innovation through Science, Kyung Hee University, Seoul 02447, Republic of Korea

[5] Kyung Hee Drug Analysis Center, College of Pharmacy, Kyung Hee University, Seoul 02447, Republic of Korea

[6] Department of Pharmacology, College of Medicine, The Catholic University of Korea, 06591, Seoul, Republic of Korea

[7] Hematology Hospital, Seoul St. Mary's Hospital, The Catholic University of Korea, Seoul, 06591, Republic of Korea

[8] Department of Biomedical and Pharmaceutical Sciences, Graduate School, Kyung Hee University, Seoul 02447, Republic of Korea

[9] Department of Pharmacy, Kyung Hee University Hospital at Gangdong, Seoul 05278, Republic of Korea

* Correspondence: ktlee@khu.ac.kr (K.-T.L.); cekchung@khu.ac.kr (E.K.C.);
Tel.: +82-2-961-0860 (K.-T.L.); +82-2-961-2122 (E.K.C.)

† These authors contributed equally to this work.

(A)

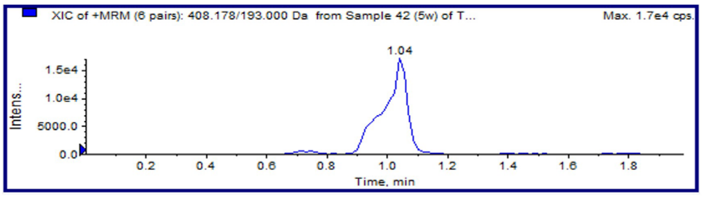

(B)

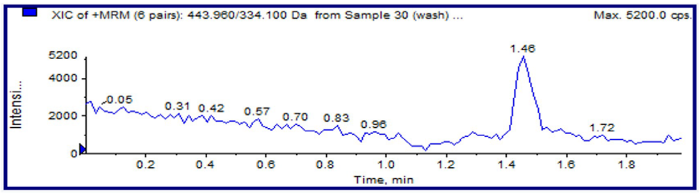

(C)

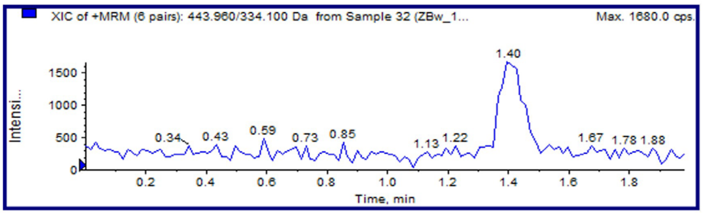

(D)

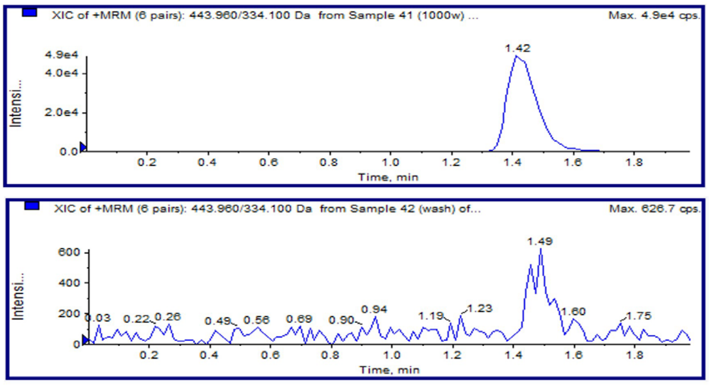

(E)

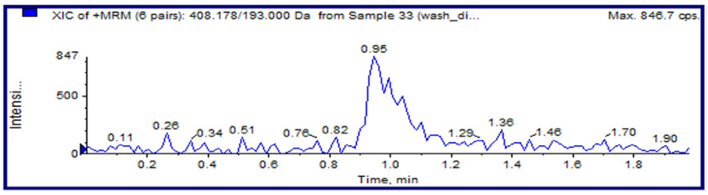

(F)

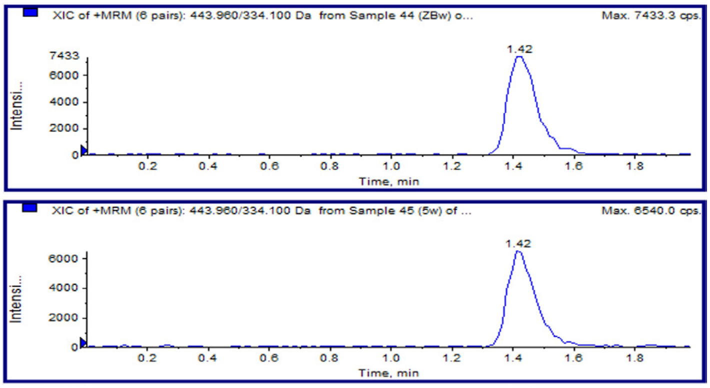

Figure S1. Representative chromatograms for selinexor and sitagliptin (internal standard [IS])

in various chromatographic conditions using the YMC-Pack C8 column [(A) to (C)] and the Luna® C18 column [(D) to (F)]. (A), simultaneous injection of selinexor (5 ng/mL) and sitagliptin (2500 ng/mL) showed the fronting and asymmetrical peak shape. (B), injection of 100% methanol as a wash solvent resulted in a baseline drift where the signal dropped from a higher intensity to a lower intensity during data collection with an unknown peak at the selinexor peak retention time. (C) injection of sitagliptin without selinexor resulted in an unknown peak at the selinexor retention time. (D) sequential injection of Sample 41 (1000 ng/mL of selinexor) followed by Sample 42 showed a small amount of carryover in the wash vial of Sample 42. (E) addition of 50% methanol as a dilution solvent resulted in an unknown peak at the IS peak retention time. (F) sequential injection of Sample 44 (zero blank, sitagliptin only) followed by Sample 45 with both selinexor (5 ng/mL, LLOQ) and sitagliptin (2500 ng/mL) resulted in a greater peak height of selinexor in Sample 44 (zero blank) compared to that in Sample 45.

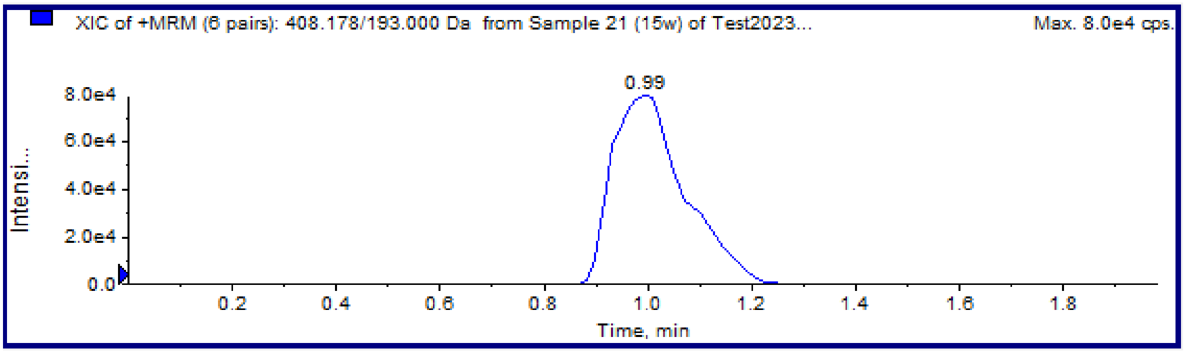

Figure S2. Representative chromatogram for selinexor in human plasma with the acetonitrile-based mobile phase showing elution of selinexor too early near the solvent front (k′ value <1).
